# Evaluation of the impact of technical physicians on improving individual patient care with technology

**DOI:** 10.1186/s12909-023-04137-z

**Published:** 2023-03-23

**Authors:** Marleen Groenier, Koen Spijkerboer, Lisanne Venix, Lars Bannink, Saskia Yperlaan, Quinten Eyck, Jeannette G. van Manen, Heleen A. Th. Miedema

**Affiliations:** 1 Technical Medical Centre, University of Twente, P.O. Box 217, 7500 AE Enschede, The Netherlands; 2 Department of Health Technology and Services Research, University of Twente, Enschede, The Netherlands

**Keywords:** Patient safety, Medical devices, Outcome assessment, Medical education, health professions

## Abstract

**Background:**

The rapid introduction of technical innovations in healthcare requires that professionals are adequately prepared for correct clinical use of medical technology. In response to the technological transformation of healthcare, a new type of professional, the Technical Physician (TP), was created and is trained to improve individual patient care using technology tailored to the needs of individual patients. This study investigates the TPs’ impact on patient care in terms of innovation, effectiveness, efficiency, and patient safety.

**Method:**

Semi-structured, in-depth interviews were conducted with 30 TPs and 17 medical specialists (MSs) working in academic or teaching hospitals in the Netherlands. The pre-structured and open-ended interview questions focused on: 1) the perceived impact on innovation, effectiveness, efficiency, and safety, and 2) opportunities and challenges in daily work.

**Results:**

TPs and MSs unanimously experienced that TPs contributed to innovation. A majority indicated that effectiveness (TP 57%; MS 71%) and efficiency (TP 67%; MS 65%) of clinical practice had increased. For safety, 87% of TPs but only 47% of MSs reported an increase. The main explanation given for TPs positive impact was combining medical and technical knowledge. Mainly organizational barriers were mentioned as a potential cause for a less visible contribution of TPs.

**Conclusion and discussion:**

TPs and MSs unanimously agreed that TPs contributed to innovating patient care through their integrative medical and technical competencies. Most TPs and MSs also reported increased effectiveness, efficiency, and safety of patient care due to the TPs’ work. TPs and MSs expected that the TPs’ impact on direct and indirect patient care will be enhanced once organizational barriers are removed.

**Supplementary Information:**

The online version contains supplementary material available at 10.1186/s12909-023-04137-z.

## Background

Many of the most important innovations in medicine are technological in nature [1]. A further increase in technology use in healthcare is anticipated [2]. Technological innovations offer opportunities for better diagnosis, treatment, and rehabilitation [3–6] however, incorrect or suboptimal medical technology use can result in inefficient care or even adverse events [7, 8]. Suboptimal use of medical technology can be traced back to technological complexity and misunderstanding of technology by users. Technology is rapidly introduced in healthcare and increasingly integrated into all aspects of clinical care characteristic of the fourth industrial revolution [9]. However, many medical technologies are black-box technologies with complex technological principles hidden from the user [10]. A lack of knowledge about these underlying technological principles is perceived as an important barrier to correct and safe use [2]. Healthcare organizations and professionals often struggle to use new technologies properly in clinical practice [11]. Many medical training programs also struggle to incorporate technological advancements into their curricula and have difficulties in preparing physicians for using medical technology (see e.g., Green et al. [12] and Hague & Merrill [13]). Medical training currently insufficiently addresses skills to integrate new technologies into daily practice. Medical education alone thus cannot address this widening gap between medicine and technology and solutions transcending disciplines are needed.

Monodisciplinary knowledge and skills no longer suffice because multiple disciplines are involved in patient care [14]. A lack of technological competence and interdisciplinary collaboration in a medical team may cause inadequate use of equipment [15] or inappropriate trust in technology [16]. In multidisciplinary healthcare teams, there needs to be an increase of effective knowledge translation between the technical and medical domain [14, 17]. Therefore, not only are changes in existing training programs required but new ways are needed for cross-disciplinary collaboration and organization of patient care. These technological developments thus require rethinking who is qualified and competent to perform procedures involving medical technology. Other healthcare professionals, such as medical physicists, have entered multidisciplinary healthcare teams to support the full clinical implementation and use of technology [18–20], changing the role of physicians. These other healthcare professionals have complementary knowledge and skills rather than specialized or extended skills. This results in blurred professional boundaries. Because these other professionals are often not legally responsible for patient care, physicians cannot just shift or delegate tasks to these new professionals. In addition, with the introduction of new medical technology, medical procedures are adjusted or completely new procedures are invented that existing healthcare professionals, such as physicians and nurses, are not trained to perform. Ensuring effective and safe technology use, therefore, requires a new type of specialized healthcare professionals, that do not need to be supervised by physicians.

In response to the increased technology use, a new hybrid technical-medical curriculum was introduced in the Netherlands: Technical Medicine. A brief summary of the educational Technical Medicine program is presented in Table 1. A detailed description of the curriculum of Technical Medicine can be found in Groenier et al. [18]. Technical Physicians (TPs) were introduced to make patient care more effective and efficient because medical technology would be used in innovative ways [21]. Trained to tailor technology use to patient-specific conditions and needs [18], the TPs work in the medical domain. They provide direct patient care, such as performing ultrasound examinations, processing scans, and assisting procedures with surgical navigation technology, but also design new clinical protocols for diagnosis and treatment involving technology (indirect patient care, see e.g., Hummelink et al. [5]). Direct patient care means they carry out interventions by having personal contact with patients. Indirect patient care means that TPs provide patient-specific care when the patient is not present, e.g., execute 3D planning of surgical interventions, contribute to multidisciplinary consultations, perform research to evaluate the effect of technical innovations in clinical practice. Indirect patient care interventions are performed away from the patient but support the overall effectiveness of those interventions. Table 2 illustrates the type of work a TP could do in clinical practice.

**Table 1:** Technical Medicine in the Netherlands

Technical Medicine is a 6-year university bachelor (undergraduate) and master (graduate) curriculum resulting in a Master of Science degree. In the first three undergraduate years, the curriculum is system-based and students acquire basic knowledge and skills concerning all human subsystems, such as the cardiorespiratory system, digestive system, urogenital system, and neural system. Cognitive integration is used to foster understanding of each system including relevant technology and guides the presentation of the information in an integrated way. Students learn about anatomy, physiology, pathophysiology, and technology for each subsystem in a systematic and structured manner. In the first year of the master, students follow technical-medical specialization courses, such as Segmentation and Visualization, Biological Control Systems, and Surgical Navigation Technology, and clinical skills and diagnostic reasoning courses covering injections, punctures and catheterizations, surgical skills, emergency reasoning, and endoscopic skills. In the two final years, students do clinical rotations in hospitals. Since 2003, over 600 Technical Physicians graduated. In 2013 a second Technical Medicine program in the Netherlands started at the Delft University of Technology in collaboration with the Erasmus Medical Center and Leiden University Medical Center. Most TPs hold research positions in hospitals. Next to their contribution to the diagnosis and treatment pathways of patients, TPs often have research and education tasks and are involved in the implementation of technical innovations. About 80% of the TPs are doing a PhD after their graduation as part of their specialization within the technical-medical domain. However, most TPs still need to follow a clinical fellowship before becoming part of the medical staff.

**Table 2:** Example of a TP working in direct patient care (graduate of 2019). The diabetic patient: personalized glucose regulation

The TP runs an outpatient clinic and sees their own patients, this mainly concerns Type 1 diabetes patients (direct patient care). These patients often have glucose sensors and insulin pumps to control their insulin levels. The TP looks at how they can improve the patient’s treatment with these technologies and adjusts the equipment to optimize patient-specific and individualized care. Together with an internist and a nurse specialized in diabetic care, the TP ensures the correct treatment of the patient. The TP is part of a multidisciplinary team that plans treatments together for specific patients. Furthermore, the TP optimizes the treatment of all diabetic patients by exploring how the programming of glucose sensors and insulin pumps can be tailored to a specific patient (indirect patient care). Case retrieved and adapted from www.nvvtg.nl	

Although TPs do not receive a traditional medical degree, they work on their own responsibility with patients (i.e., they do not require supervision by a physician) and are legally recognized and qualified to perform specified reserved medical procedures in the Netherlands [18, 21]. However, there are still some barriers that might limit which procedures and interventions they can perform in clinical practice which in turn might affect their impact on direct and indirect patient care [21]. This could be explained by the novelty of the discipline and the lack of a shared understanding of this new discipline. Emerging professions often lack recognition of their expertise by existing professionals which causes challenges in performing their daily work [22]. In the case of TPs, this might affect their opportunities to make an impact in direct patient care. A Dutch national study by De Haan et al. [23] already showed that most TPs did perform specified reserved medical procedures regularly, but they did not investigate whether the TPs’ work contributed to improving direct and indirect patient care. In this paper, we explore the impact of TPs on direct and indirect patient care.

### Research objective

A transformation of healthcare is needed because of societal and technological challenges [24]. According to the Institute of Medicine [24], the healthcare system should be safe, effective, patient-centered, timely, efficient, and equitable. TPs are trained to use technology in innovative ways to make healthcare safer, more effective, and more efficient, which is in line with the quality dimensions proposed by the Institute of Medicine. In 2009, the first students graduated from the Technical Medicine program. To our knowledge, the TPs’ impact on clinical practice has not yet been investigated. Our primary research question is: How do TPs and medical specialists (MSs) perceive the contribution of TPs to innovative, effective, efficient, and safe medical technology use in both direct and indirect patient care? Our secondary research question is: To what extent do TPs experience barriers and facilitators in their daily work as an emerging profession? This study provides insight into how specifically trained high-tech healthcare professionals (TPs) might complement existing professions in healthcare.

## Methods

### Study design and setting

Since the research questions were explorative and concerned the experiences of stakeholders, semi-structured, in-depth interviews were considered most appropriate [25]. TPs and MSs from academic and teaching hospitals in The Netherlands were interviewed between 2017 and 2019. This study was exempt from medical ethical review in the Netherlands (IRB East Netherlands Arnhem-Nijmegen, file number 2022–13,876) because this research was not subject to the Dutch Medical Research Involving Human Subjects Act (WMO). University regulations in effect during the study period (2017–2019) specifically excluded projects from requiring ethical approval, as no University Research Ethics Policy existed at that time. The research was carried out in accordance with the Declaration of Helsinki. Informed consent was obtained from all participants and confidentiality was guaranteed.

### Study population and recruitment

At the time of the study, over 500 TPs had graduated and according to information from the Dutch Association for Technical Medicine (NVvTG) the majority of TPs worked in a medical center (about 75%), mostly in a PhD position (80%). Other TPs were employed at universities or healthcare companies. We contacted TPs through the Technical Medicine program of the University of Twente and the NVvTG. TPs could participate if they were involved in direct or indirect patient care and worked at a clinical department in an academic or teaching hospital. We aimed for a diverse sample of TPs in terms of experience, medical specialty, technology, type of care (diagnosis, treatment, or aftercare) and from different hospitals across the Netherlands. None of the TPs who agreed to participate were excluded. TPs who participated were asked for the contact information of the MSs who supervised or worked together with them. MSs were subsequently contacted and asked to participate. After data collection, one of the research assistants removed identifying information about the participants and assigned identification numbers.

### Interviews

The pre-structured and open-ended interview questions were divided into two categories: impact indicators (innovation, effectiveness, efficiency, and safety) and opportunities and challenges in daily work (see Appendix A for the interview guide). TPs were asked about the impact indicators and opportunities and challenges in daily work. MSs were only asked about the impact indicators. Six research assistants, five of whom were part of the research team, conducted the interviews in person or by video call and all interviews were audio-recorded. Research assistants conducted the interviews because they were more impartial to the outcome of the study than the other team members.

### Data analysis

In this study, a thematic content analysis was used. For the first research question also a framework analysis approach was used. Each interview was reviewed by a different pair of research assistants. The first research assistant categorized whether a participant mentioned ‘increase’, ‘no increase’, or ‘unable to assess’ for each impact indicator. After that, a list of codes was created for each impact indicator based on the statements from the interviews. The second research assistant reviewed this work and revised the categorization and codes, based on the original recordings. Next, three research assistants categorized the codes into broader themes for each impact indicator across all TPs and MSs. In case of disagreements between the research assistants, differences were discussed until a consensus was reached. The analysis resulted in 1) the number of TPs and MSs who reported ‘increase’, ‘no increase’ or ‘unable to assess’, 2) a set of codes for each category for each of the impact indicators, and 3) a set of opportunities and challenges in clinical practice reported by TPs.

### Reflexivity statement

All authors adopted a reflexive attitude to discussing their initial observations of the data in relation to their own interests and biases. Six out of eight authors were actively involved in the Technical Medicine program as students or faculty and discussed the potential biases and presuppositions resulting from this involvement. They considered their own (occupational) roles and how these might affect their initial reading of the data. At the time of the data analysis, MG was a senior researcher at the Technical Medical Centre with experience in technical-medical simulation education. KS, LV, LB, and QE were TPs in training. SY was a biomedical engineer in training. JvM was a senior researcher with experience in health program evaluation. HM was the curriculum designer and director of education of Technical Medicine.

## Results

A total of 30 TPs and 17 MSs from different departments of seven medical centers in The Netherlands participated. The TPs had on average 4 years of work experience which ranged from a few months to 9 years. Of the 30 TPs, 19 held a permanent position. About half of the TPs held a research position (*n* = 16) while twelve TPs held a TP or TP fellow position. Two TPs held an ‘other’ position. All MSs were senior faculty members (*n* = 11) or chief physicians (*n* = 6) and worked with one or more TPs daily.

### Impact of TPs on clinical care

TPs and MSs were asked to what extent TPs contributed to innovation, effectiveness, efficiency, and safety of direct and/or indirect patient care. Table 3 shows the number of TPs and MSs who perceived an increase in innovation, effectiveness, efficiency, and safety, no increase, or were unable to assess the impact of the TPs’ work. Below we describe the associated codes for each indicator and the frequency a code was assigned. The coding was not mutually exclusive: a particular participant could mention different aspects for one impact indicator. We provide illustrative quotes for each of the impact indicators. Also, TPs’ perceived opportunities and challenges are described.

**Table 3:** Number and percentages of Technical Physicians and medical specialists for the four impact indicators

	**Increase**		**No increase**		**Unable to assess**	
	*n*	%	*n*	%	*n*	%
**Innovation**						
Technical physician	30	100	0	0	0	0
Medical specialist	17	100	0	0	0	0
**Effectiveness**						
Technical physician	17	57	1	3	12	40
Medical specialist	12	71	0	0	5	29
**Efficiency**						
Technical physician	20	67	3	10	7	23
Medical specialist	11	65	2	12	4	23
**Safety**						
Technical physician	26	87	1	3	3	10
Medical specialist	8	47	6	35	3	18

Technical physicians (*n* = 30) and medical specialists (*n* = 17) who perceived an increase, no increase, or were unable to assess the impact of the TP’s work for the four impact indicators in The Netherlands between 2017–2019

### Innovation

TPs owed their contribution to innovating healthcare to their specific technical-medical competencies (TP: *n* = 10). TPs also attributed their contribution to their scientific approach to patient care (TP: *n* = 2) which is in line with one MS who mentioned that an academic setting and available funding played an important role (MS: *n* = 1). In addition, participants mentioned the TPs’ responsibility for implementing an innovation (TP: *n* = 7) and improvement of clinical and technical processes through the TPs’ work (TP: *n* = 1):

*“TPs can easily switch between new technological developments and their application for and translation to the individual patient population. They have the creativity and expertise to evaluate new safe applications for a particular technique or drug.”* TP-14

### Effectiveness

About half of the TPs reported an increase in effectiveness (*n* = 17) which was evidenced by a higher quality of care according to seven TPs. Also, participants mentioned various reasons for an increase in effectiveness: the introduction of new or better (non-invasive) techniques (TP: *n* = 1; MS: *n* = 2), lowering chances of complications (TP: *n* = 1), or enabling earlier hospital discharge (TP: *n* = 1). Six TPs mentioned improved clinical processes. Clinical processes were improved because of earlier diagnosis (TP: *n* = 1), multidisciplinary consultations (TP: *n* = 1), bringing in imaging and performing therapies (TP: *n* = 1), saving operating time through preoperative planning (TP: *n* = 1), being the contact person in a clinical process that transcends departments and disciplines (TP: *n* = 1), or the TPs’ versatility (MS: *n* = 1). The TPs’ work also resulted in clinical processes that were more tailored to the patients’ needs (TP: *n* = 5), e.g., by better preoperative planning and patient consultations (MS: *n* = 3), and improved diagnostics (MS: *n* = 2):

*“Often, as a TP, you have a clear role in individualizing the treatment, where you try to better tailor the treatment to the individual patient. Through quality checks, we aim to move towards a more personal treatment plan. We can then better assess during the procedure whether it has been effective and whether further treatment is necessary so that we do not under-treat during the procedure. This means avoiding the need for re-treatment at a later date.”* TP-30

*“I really think they have a big contribution to diagnostics, which we didn't have before, so that treatment is carried out more effectively and much more specifically based on the measurements and information that the TP provides.”* MS-03

Four TPs explained that they expected an increase in effectiveness but that there is no scientific evidence to support that conclusion yet.

Here too, the work of some TPs focused on research which made it difficult to assess the TPs’ impact (TP: *n* = 8; MS: *n* = 2). Two TPs mentioned that their research had the potential to influence effectiveness in the future. One TP mentioned a change in the efficiency of patient care but could not yet detect an increase in effectiveness. Three MSs found it difficult to ascribe the increase in effectiveness to only the TPs’ work. In contrast with the impact on efficiency, more TPs and MSs stated that they could not adequately assess the impact of the TPs’ work on patient care because the TPs’ work focused on research.

### Efficiency

Participants attributed an increase in efficiency to the TPs’ ability to apply technical and medical competencies in individual patient care (TP: *n* = 12; MS: *n* = 6) resulting in a shorter duration of patient interventions (TP: *n* = 9) and more accurate interventions (TP: *n* = 3):

*“We are increasingly moving towards a better selection of patients who actually benefit from the intervention, and my research also aims to see if it is possible to get more information from data that we already have. I set up this innovation together with a medical specialist here, and a Technical Medical student is also participating in the research.”* TP-04

*“Due to the arrival of the TP, we have really started treating our patients differently. This is one of the first TPs to work here, they have set everything up and are also actually part of the care process. [...] In this sense, this saves the specialist time and avoids unnecessary surgery being carried out, which has made the operations more efficient, but also more predictable.”* MS-11

However, some participants reported that it was hard to assess the impact of TPs’ work on efficiency (TP: *n* = 2; MS: *n* = 2). One MS stated that increasing efficiency is not the primary purpose within their medical field and one MS stated that there was a lack of scientific evidence showing the impact on efficiency. For some TPs the work mainly focused on research, making it more difficult to assess TPs’ impact on the efficiency of patient care (TP: *n* = 6; MS: *n* = 4):

*“The TP here is a researcher and in that way, the knowledge about equipment has increased due to the presence of the TP and a connection has been made. The TP only researches new equipment and therefore does not support us with current equipment. [....] The new applications still have to prove themselves, but I do expect it to work and that it will give us an edge as a result.”* MS-04

### Safety

The TPs ascribed an increase in safety, among others, to their technical-medical expertise (TP: *n* = 9), overseeing safety in clinical processes (TP: *n* = 6), the application of safety margins and outcomes (TP: *n* = 3), and preventing potential problems and risks during procedures (TP: *n* = 5):

*“In the field of safety, big steps are being made because the TP clearly understands what can go wrong. For example, what to look out for with equipment, why a certain alarm goes off, where that comes from, how to prevent it, and improve safety margins. I think that really improves safety. This is important for the safe application of new techniques. This allows you to do this faster and more safely compared to someone who has studied Medicine.”* TP-15

The MSs who noticed an increase mentioned different reasons for this increase, e.g., because TPs had more knowledge about technical equipment use (MS: *n *= 2), they were keeping track of risks associated with technology use (MS: *n *= 1), and they were able to apply the right therapy sooner due to improved diagnostics (MS: *n *= 1).

*“Yes, perhaps indirectly, because there are all kinds of new techniques being used, the TP is aware of the pitfalls of these techniques. This, therefore, contributes to safety.”* MS 09

Some TPs and MSs could not determine whether the TPs’ work specifically contributed to patient safety (TP: *n* = 3; MS: *n* = 3). Other reasons mentioned by the participants for a lack of impact on safety were that safety precautions can also be guaranteed by other specialists (TP: *n* = 1), increased safety was a group effort (MS: *n* = 1), increased safety could be achieved in the future, but was now limited (MS: *n* = 6), or that they were unable to assess the impact of the TPs’ work on patient care (TP: *n* = 4; MS: *n* = 3). One TP mentioned that they did not contribute directly to the safety of a procedure.

### Opportunities and challenges in clinical practice

As an emerging profession, TPs might experience opportunities and challenges in clinical practice. Most TPs hold a research position, either as part of their specialization as TP or because there is no appropriate clinical job profile available yet (see also Table 1). In the current study, the TPs were asked about the opportunities and challenges they experienced in clinical practice. Eighteen TPs reported experiencing many possibilities and freedom within their job profile in the hospital, such as freedom for personal development (*n* = 3), managing their own research (*n* = 5), and defining and managing daily tasks themselves (*n* = 9). Ten TPs explicitly mentioned they worked without supervision in patient care. TPs indicated that depending on their job profile in the hospital, they could apply their research insights to patient care (*n* = 4) and use their technical-medical expertise (*n* = 4).

Over half of the TPs (*n* = 19) encountered limitations in daily clinical practice, while 11 TPs reported experiencing no limitations. They explained that because of a lack of a uniform and formal job description, they experienced limitations in performing patient care without supervision and with legal responsibility for their own patients, although this is something they were specifically trained for. The limitations they encounter are, e.g., inaccessibility to patient files (*n* = 7), unfamiliarity with the job profile (*n* = 9), and no authorization for clinical activities (*n* = 4).

## Discussion

There is a growing need for specifically trained high-tech healthcare professionals to reduce the misuse of medical technology and improve the quality of care of patients using technology tailored to the needs of individual patients. Hybrid curricula at the intersection of multiple disciplines, such as the Technical Medicine program, could potentially bridge the gap between medicine and technology [20, 26]. In the current study we examined how TPs and the MSs they worked with perceived the contribution of TPs to patient care in terms of innovation, effectiveness, efficiency, and safety and to what extent TPs experience barriers and opportunities in clinical practice.

All TPs and MSs experienced an increase in innovation and most of them experienced an increase in efficiency and effectiveness of patient care because of the TPs’ activities. This positive impact was mainly attributed to the TPs’ unique ability to combine technical and medical competencies in patient care. The TPs’ activities in patient care resulted in improved use of new or existing technology with individual patients (efficiency) and improved quality of patient care, reduced risks associated with technology use, and optimized clinical workflow (effectiveness). While most TPs observed an increase in safety because of increased awareness of risks associated with medical technology use, about half of the MSs did not see an increase in safety due to the involvement of a TP. Also, most TPs experienced possibilities and freedom to shape their own tasks and job profile, even though some TPs experienced organizational barriers.

Although overall TPs and MSs were positive about how TPs’ work affected patient care, some TPs and MSs reported they perceived no impact on efficiency, effectiveness, or safety or found it difficult to assess the impact. The main reasons mentioned by TPs and MSs for this lack of impact were the absence of scientific evidence about the impact of the TPs’ work on patient care and that TPs fulfilled a research position instead of a clinical position in the hospital. TPs can be involved in patient care in various ways depending on the type of care pathway (diagnosis, treatment, or aftercare), the type of technology used, and whether their work is related to direct or indirect patient care. Although TPs are specifically trained to provide patient care without the supervision of a physician, which fits with a clinical position, TPs in a research position can also make an impact if they are part of a clinical department or medical team. Whether a TP holds a clinical or a research position does seem to influence their opportunities to carry out direct patient care activities as they were trained to do, i.e., diagnose and treat individual patients with technology.

The challenges that TPs experienced in clinical practice were often related to direct patient care activities. Possibly the lack of recognized expertise for this emerging profession in combination with a lack of a uniform job profile [22] limits their opportunities to apply their technical-medical expertise and practice medicine independently. This resonates with the development of other emerging professions, such as nurse practitioners [27, 28] and physician assistants [29]. Van den Brink et al. [29] described how physician assistants grew from being a substitute for a physician in a protocol-driven context to executing tasks independently and being recognized for having complementary skills. Similar to the experience of the TPs in our study, supervision of physician assistants by physicians was considered a necessary activity, especially in the first decade of the introduction of the profession [29]. According to De Bont et al. [30] advances in new technologies and managerial practices result in extended professional roles, such as those of nurse practitioners and physician assistants. In line with our study and Van den Brink et al.’s review, [29] De Bont et al. [30] show that professionals with new, extended roles or from emerging professions are dependent on legally defined scopes of practice, personal relationships between healthcare professionals, and trust in their competences. This is even more challenging for TPs because their work focuses on innovation which means they adapt and change standards and protocols. TPs were recently introduced in the Netherlands and they are confronted with similar barriers and facilitators common to other emerging professions in healthcare that were introduced in other countries over the past decades.

We observed a clear discrepancy in the perception of TPs and MSs about the impact of the TPs on patient safety. MSs more frequently mentioned that maintaining patient safety while working with medical technology is a team effort and responsibility. This indicates that MSs have a systems perspective on patient safety. Patient safety is determined by many other systemic factors apart from the appropriate use of technology [31]. For future research, it is worth disentangling the contribution of various actors to maintaining patient safety and how they interact with each other and with the technological tools they use. Insight into how different disciplines work together could aid in identifying learning points for medical technology use in clinical practice, a better definition of the role of the TP in multidisciplinary teams, and is essential for continuing professional education of physicians aimed at multidisciplinary teamwork [32, 33].

Hybrid professionals trained in technical-medical curricula are proposed as one solution to counter the adverse effects of rapid technology development and introduction in healthcare [18, 20, 26]. Our study shows tentatively that TPs who were trained in a hybrid curriculum aimed at integrating technical and medical competencies positively contributed to improving patient care. To our knowledge, the Technical Medicine program is the first academic curriculum that was specifically designed with the aim of technical-medical expertise development. Also, this is the first study evaluating the perception of the impact of TPs on innovating patient care using technology. Groenier et al. [18] explain that for adequate medical technology use with individual patients, healthcare professionals need specific technical-medical expertise. The participants in our study frequently mentioned the ability to integrate technical and medical competencies as a unique and distinctive characteristic of the graduates from the Technical Medicine program.

### Strengths & limitations

Our study design had some limitations. First, the sample of interviewed TPs might not be representative of all graduated TPs working in medical centers because only TPs working at academic or teaching hospitals were included. However, our sample consisted of a diverse group of TPs with different affiliations, work experience, and positions held. TPs working in different contexts, e.g., medical-technical companies or outpatient care, should be included in future studies to broaden the perspective about the impact TPs can have on healthcare.

Second, social desirability bias might have occurred because our data were self-reported. The impact was reduced by including both the perspective of the TP and the MS. Our results showed that TPs’ and MSs’ perspectives mostly agreed. Our sample of TPs and MSs was self-selected, which might also introduce bias in our results. MSs who are working with TPs might be more positive in their perception of the impact of TPs. In our exploratory study, we aimed for a first orientation on the possible impact of the TPs’ work. Further studies are needed for a more in-depth analysis as well as the specification of the contexts which help or hinder TPs to innovate patient care.

Third, most team members of the research team were actively involved in the Technical Medicine program which on the one hand might have influenced the interpretation of the interviews. On the other hand, the interviewers’ experience within the field of Technical Medicine allowed for in-depth follow-up questions.

### Future directions and practical implications

We described some issues in understanding how professionals of an emerging, hybrid profession in healthcare can make an impact on improving patient care using technology. One of the issues mentioned by our participants was that there was a lack of scientific evidence about the impact of the TPs’ work. A mix of qualitative and quantitative methods and a more diverse group of stakeholders, e.g., hospital administrators and patients, is needed to achieve a more detailed understanding of the impact of TPs on healthcare [27–29].

Previous research on the implementation of nurse practitioners in healthcare showed that interpersonal and teamwork skills seem essential for the acceptance of an emerging profession by existing professions [27, 28]. Further research is therefore also needed on how these skills affect the TPs opportunities to be involved in direct patient care and gain trust in their competences. To prepare TPs for their role in clinical care, Technical Medicine curricula should pay explicit attention to professional identity development, professional attitude, and professional skills such as communication, teamwork, and personal leadership. These competencies are likely needed for TPs to gain trust while collaborating with other healthcare professionals, as evidenced by studies on the implementation of nurse practitioners [27, 28].

Furthermore, multidisciplinary collaboration is increasingly important for healthcare professionals to properly implement technical innovations and use technology in a safe, effective, and efficient way. Continuous professional education of not only TPs but also physicians, nurses, and other allied healthcare professionals should therefore focus on skills that support multidisciplinary collaboration. In multidisciplinary teams, professionals’ expertise is often complementary, and learning about the various disciplinary perspectives on patient care could aid improved collaboration in practice.

## Conclusion

According to most medical specialists and Technical Physicians, professionals with dedicated technical-medical training (TPs) successfully applied their knowledge and skills to drive innovation and increase the efficiency and effectiveness of patient care. Based on our results we conclude that the TPs’ impact on direct patient care could be more pronounced if the organizational infrastructure allowed TPs to practice medicine independently. This would mean that they are responsible for their own clinical activities without supervision and can be held accountable for their actions by Dutch disciplinary law. We conclude that TPs trained in the Technical Medicine program complement existing expertise in medical teams to fulfill the urgent unmet need of technology-competent healthcare professionals to make healthcare more innovative, efficient, effective, and to a lesser degree also safer.

## Supplementary Information

**Additional file 1.** Appendix A

## Data Availability

The datasets used and/or analyzed during the study are available from the corresponding author upon reasonable request.
