# Supplementary material for: Evaluation of the impact of technical physicians on improving individual patient care with technology
Source: BMC Med Educ. 2023 Mar 23;23:181. doi: 10.1186/s12909-023-04137-z (PMC10037766; doi:10.1186/s12909-023-04137-z)
Supplement: Supplementary file 1 — Additional file 1. Appendix A [file 12909_2023_4137_MOESM1_ESM.docx]

Appendix A: Interview guide

Interview guide Evaluating impact Technical Physician in clinical care

**Questions for Technical Physician**

**Activities TP**

- 1. 1. In which track did you graduate: Medical Imaging & Interventions, Medical Signals & Systems, or Reconstructive Medicine?
  2. 2. What was the date of your graduation?
  3. 3. In which department are you currently employed?
  4. 4. Who is your superior?
  5. 5. What is your job profile?
  6. 6. Is your contract temporary or permanent, and how is it funded?
  7. 7. Which barriers and opportunities do you experience in your current job profile?
  8. 8. Which barriers and opportunities do you experience in carrying out medical interventions including reserved procedures?
  9. 9. Can you describe your role in the care pathway? Which patient population are you involved in? Could you describe the interventions you carry out in this care pathway?

**Impact TP**

10.To what extent does the presence of a TP in your department increase efficiency?

For the interviewer: Provide examples if necessary such as the use of equipment, duration of intervention, and follow-up appointments.

10a. Please rate the increase in efficiency on a scale from 1 to 5 with 1 being no increase and 5 large increase.

11. To what extent does the presence of a TP make the care pathway more effective?

For the interviewer: Provide examples if necessary such as targeted diagnosis/treatment, patient comfort, and complications.

11a. Please rate the increase in effectiveness on a scale from 1 to 5 with 1 being no increase to 5 a large increase.

12. To what extent does the presence of a TP increase innovation in your department?

13. To what extent does the presence of a TP increase patient safety in your department?

13a. Does the presence of a TP in your department prevent medical error?

14. Do you have any further remarks or are there topics you would like to address?

**Questions for the medical specialist**

1. Could you describe your job profile?

**Impact TP**

1. 2.To what extent does the presence of a TP in your department increase efficiency?
2. For the interviewer: Provide examples if necessary such as the use of equipment, duration of intervention, and follow-up appointments.
3. 2a. Please rate the increase in efficiency on a scale from 1 to 5 with 1 being no increase and 5 large increase.
4. 3. To what extent does the presence of a TP make the care pathway more effective?
5. For the interviewer: Provide examples if necessary such as targeted diagnosis/treatment, patient comfort, and complications.
6. 3a. Please rate the increase in effectiveness on a scale from 1 to 5 with 1 being no increase to 5 a large increase.
7. 4. To what extent does the presence of a TP increase innovation in your department?

5. To what extent does the presence of a TP increase patient safety in your department?

5a. Does the presence of a TP in your department prevent medical error?

1. 6. To what extent does the presence of a TP in your department have a positive effect on the reputation of your department?

6a. If there is a positive effect: how does that show?

For the interviewer: Please provide examples if necessary such as more patients, more publications, and invited talks.

Performing reserved procedures

7. TPs are legally allowed to perform certain reserved procedures. In your opinion, is the current list sufficient for the TPs in your department to perform their job? Are any procedures missing that you consider to be required for a TP?

8. If the TP would not be able to independently practice medicine, in what way would this affect clinical care in your department?

For the interviewer: This could be in terms of efficiency, effectiveness, innovation, and safety.

9. If there were no TP in your department, what would you miss?

Activities TP:

10. How is the position of the TP in your department funded?

11. Can you retain the TP in your department? Could you explain why/why not?

1. 12. Do you have any further remarks or are there topics you would like to address?
